# The effect of age on the induction dose of propofol for general anesthesia in dogs

**DOI:** 10.1371/journal.pone.0288088

**Published:** 2023-07-03

**Authors:** Chiara E. Hampton, Anderson da Cunha, Amber Desselle, Patricia Queiroz-Williams, Erik H. Hofmeister

**Affiliations:** 1 School of Veterinary Medicine, Department of Veterinary Medical Science, Louisiana State University, Baton Rouge, Louisiana, United States of America; 2 College of Veterinary Medicine, University of Tennessee, Knoxville, Tennessee, United States of America; 3 College of Veterinary Medicine, Midwestern University, Glendale, Arizona, United States of America; 4 College of Veterinary Medicine, Department of Clinical Sciences, Auburn University, Auburn, Alabama, United States of America; Universidade de Trás-os-Montes e Alto Douro: Universidade de Tras-os-Montes e Alto Douro, PORTUGAL

## Abstract

**Objective:**

In people, the dose of propofol (DOP) required for procedural sedation and anesthesia decreases significantly with age. The objective of this study was to determine if the DOP required to perform endotracheal intubation decreases with age in dogs.

**Study design:**

Retrospective case series.

**Animals:**

1397 dogs.

**Methods:**

Data from dogs anesthetized at referral center (2017–2020) were analyzed with three multivariate linear regression models with backward elimination using a combination of either absolute age, physiologic age, or life expectancy (ratio between age at the time of anesthetic event and expected age of death for each breed obtained from previous literature) as well as other factors as independent variables, and DOP as the dependent variable. The DOP for each quartile of life expectancy (<25%, 25–50%, 50–75%, 75–100%, >100%) was compared using one-way ANOVA. Significance was set at alpha = 0.025.

**Results:**

Mean age was 7.2 ± 4.1 years, life expectancy 59.8 ± 33%, weight 19 ± 14 kg, and DOP 3.76 ± 1.8 mg kg^-1^. Among age models, only life expectancy was a predictor of DOP (-0.37 mg kg^-1^; *P* = 0.013) but of minimal clinical importance. The DOP by life age expectancy quartile was 3.9 ± 2.3, 3.8 ± 1.8, 3.6 ± 1.8, 3.7 ± 1.7, and 3.4 ± 1.6 mg kg^-1^, respectively (*P* = 0.20). Yorkshire Terrier, Chihuahua, Maltese, mixed breed dogs under 10 kg, and Shih Tzu required higher DOP. Status of neutered male, ASA E, and Boxer, Labrador and Golden Retriever breeds decreased DOP, along with certain premedication drugs.

**Conclusions and clinical relevance:**

In contrast to what is observed in people, an age cut-off predictive of DOP does not exist. Percentage of elapsed life expectancy along with other factors such as breed, premedication drug, emergency procedure, and reproductive status significantly alter DOP. In older dogs, the dose of propofol can be adjusted based on their elapsed life expectancy.

## Introduction

Propofol is a widely used hypnotic drug able to induce and maintain general anesthesia in people and animals [1, 2]. Propofol inhibits the N‐methyl‐D‐aspartate (NMDA) receptor and activates the ionotropic subtype of the γ‐aminobutyric acid receptor-A (GABA_A_), causing hyperpolarization of the postsynaptic cell membrane and a dose-dependent depression of the central nervous system (CNS) [3]. In dogs, as in people, propofol is commonly used for procedural sedation, and induction and maintenance of general anesthesia [4].

Dogs are heavily relied upon as comparative biomedical research models for several human physiologic and pathophysiologic processes, including aging [5, 6]. In fact, several literature reviews have been published in regard to the usefulness of pet dogs as longitudinal research model for aging and age-associated morbidity and mortality [5–9]. In the last decades, the average life span of dogs has progressively increased. Roughly 30% of the population of client-owned dogs is nowadays considered geriatric [10]. Lower anesthetic requirements of many analgesic and hypnotic drugs in elderly patients, including propofol, have been observed in people [11–15].

There is a large body of literature investigating the relationship between the dose of propofol (DOP) and age in people. It has been shown that, not only is the DOP for induction of a person 60 years of age and older (1.5–1.75 mg kg^-1^) significantly lower than for people under 60 years (2.25–2.5 mg kg^-1^), but also that elderly patients receiving a DOP above 1.75 mg kg^-1^ would manifest significant hypotension and apnea [15]. In 1998 and 1999, two capstone works published in *Anesthesiology* demonstrating the influence of age on the pharmacokinetics and pharmacodynamics of propofol showed that aging affects the intercompartmental distribution of propofol and increases brain sensitivity to propofol [16, 17]. More recent clinical studies have reported that elderly patients (≥65 year-old) receiving propofol in the emergency department required a significantly lower dose for induction and maintenance of procedural sedation compared to young adults [18], and that when doses higher than the recommended one were used for this age group, the incidence of post-induction hypotension was greater [19]. Furthermore, age is one of the factors included in a published formula that predicts the dose of propofol to prevent post-induction hemodynamic fluctuations in human patients [20].

Even though this pharmacological variance is well documented in the geriatric human population, studies have yet to elucidate if a comparable phenomenon occurs in elderly dogs (*Canis lupus familiaris*). The assumption that aging may also affect DOP in the canine elderly population as observed in people with similar adverse effects is not an unreasonable one. The incidence and magnitude of adverse events after propofol administration, such as post-induction decrease in systolic arterial pressure [21], hypotension [22], and apnea [23], are dose-dependent and more accentuated in the human elderly, and the anesthesiologist may modify dosing with the goal of optimizing post-operative outcomes [19]. In elderly canine patients, the incidence of adverse events after propofol administration is unknown. Post-induction dose-dependent apnea [24] and decrease in mean arterial pressure [25–27] are common occurrences in dogs administered propofol for induction of general anesthesia. Hence, it is logical to infer that the lowest possible DOP should be used to accomplish induction of general anesthesia in order to minimize these potential adverse effects.

The primary goal of this study was to determine the effect of aging on the intravenous (IV) DOP required to induce general anesthesia and facilitate endotracheal intubation in dogs, whereas the secondary goal was to identify other predictors of DOP. The tested null hypothesis stated that the DOP per unit of mass required to perform endotracheal intubation in elderly dogs would not differ from that required for young and adult subjects, and that none of the investigated variables would constitute a predictor of DOP.

## Materials and methods

This study was designed as a retrospective case series study including demographic and perianesthetic data collected from dogs that underwent general anesthesia from January 2017 to June 2020 at the Anesthesiology Service of the Louisiana State University, Veterinary Teaching Hospital. Informed owner consent to general anesthesia and for data collection was obtained from all owners prior to the anesthetic event. The Louisiana State University Institutional Animal Care and Use Committee (LSU IACUC) was consulted prior to the start of this study about the need for their oversight. We obtain verbal and written confirmation from the LSUIACUC that retrospective studies using historically collected data or tissues does not require their oversight in keeping with national and international standards.

### Data collection

A digital anesthetic record (DAR) software (VetDAR, Dimple Hill Software LLC, Corvallis, OR) was used to collect and store canine patient demographic and anesthetic-related data. According to the anesthesiology service’s standard operating procedure, a senior veterinary student created and maintained an electronic anesthetic record for each anesthetic event and dog, generating a unique record. After dogs had recovered from anesthesia and records were completed by senior students, these were checked for accuracy by the technician assigned to the clinical case, and subsequently by the anesthesiologist on duty. Aesthetic records were then approved and locked to prevent *post-hoc* modification and tampering. An Excel (Version 16.65 2022; Microsoft Corporation, Redmond, WA) spread sheet including all anesthetic cases contained into the database of DAR was generated. All anesthetic records collected between January 2017 and June 2020 were included in the study. Further case selection and filtering was carried out by including only cases containing the terms “Canine” under the category “Species”, and “Propofol” under the “Induction Agent” used. Dogs receiving propofol (Propoflo; 10 mg mL^-1^, Zoetis Inc., Kalamazoo, MI) as part of their induction regimen were included in the studied cohort (*n* = 2346). If demographic data were missing from the anesthetic record file, they were retrieved from the digital hospital managing software (Cornerstone, IDEXX, Westbrook, ME). If data was irretrievable or inaccurate, the anesthetic record was excluded from the study (*n* = 924). The same investigator performed data gathering and filtering to handle the data uniformly. Dogs receiving remifentanil as a constant rate infusion as a co-induction agent with propofol were excluded from the study (*n* = 25).

### Propofol administration

Sedation prior to induction of general anesthesia was subjectively scored by a veterinary student based on a 4-point descriptive scale (0 = no sedation; 1 = mild sedation; 2 = moderate sedation; 3 = profound sedation). Administration of propofol prior to endotracheal intubation was performed by manual injection, starting from a standard estimated dose of 4 mg kg^-1^, which was then slowly (at an approximate rate of 1 mg kg^-1^/30 seconds) titrated with the end points of loss of medial palpebral reflex and of jaw tone before performing endotracheal intubation. Propofol injections were performed by senior veterinary students closely supervised by a veterinary anesthesia technician, anesthesia intern, anesthesia resident, or anesthesiologist on duty. After intubation was performed and maintenance of general anesthesia started, the total DOP (mg) was recorded on DAR. The dose per unit of mass was automatically calculated by dividing the total administered dose by the weight of the dog at the time of anesthesia.

#### Independent variables

*Age*. For statistical analysis purposes, three variants of the age concept were investigated as the independent variables of the statistical analysis model:

1. Absolute age (year): Calculated in days by subtracting the date of birth from the date of procedure and then converted into year by dividing the number of days by 365.
2. Elapsed percentage of life expectancy (percentage): Life expectancy was defined as the average period of time that a dog of a specific breed is expected to live. As substantial breed variation in longevity exists in the canine species, despite apparent similarities in physiologic, dietary, and environmental factors [28–30], life expectancy of each breed (in days) was calculated by averaging the median values of life expectancy for each breed from the available literature [30–34], and percentage of elapsed life expectancy calculated for each subject based on the age at the time of the anesthetic episode. Dogs were then allocated in 5 interquartiles representing 0–25%, 26–50%, 51–75%, 76–100%, >100% of their expected life expectancy. Toy and miniature sizes of Poodles and Schnauzers were grouped together, and average median life expectancy was calculated for the two groups.
3. Physiologic age (year): Defined as the chronological age of a canine subject corresponding to human equivalent based on a polynomial curves relating human age to canine age constructed for different sizes of dogs (weight ranges: 6.8–13.6, 13.7–22.7, 22.8–34, 34.1–45.3, >45.4 kg) [35].

#### Primary outcome measure

*Dose of propofol*. mass of propofol (mg) required to perform endotracheal intubation per unit of mass (kg) of the dogs included in this study.

#### Secondary outcome measures

*Demographic and physiologic variables*. From the subset of dogs receiving propofol, alone or in combination with other IV hypnotic, dissociative, and/or sedative agents for induction of anesthesia, the following data were extracted from DAR: body weight, body condition score (BCS), sex, reproductive status, and breed. If the words “Mix”, “Mixed”, or “Cross” was present in the assigned breed name, dogs were allocated into two mixed-breed groups according to their body weight (< 10 kg and >10 kg, respectively) according to previously published data for purpose of data analysis [32].

*Perianesthetic data*. Date and type of procedure (5 categories: dental, soft tissue surgery, orthopedic surgery, imaging, minimally invasive procedure), American Society of Anesthesiologists physical and emergency status (ASA E) [36], premedication regimen used, level of sedation after premedication, route of administration of premedication were obtained as defined in Table 1.

**Table 1:** Variables and score system for demographic and pre-anesthetic data evaluating the influence of age on the induction dose of propofol in dogs.

Variable	Score	Description
Body Condition Score	1	Emaciated
	2	Very thin
	3	Thin
	4	Underweight
	5	Ideal weight
	6	Overweight
	7	Heavy
	8	Obese
	9	Severely obese
Level of sedation after premedication	1	None
	2	Mild
	3	Moderate
	4	Severe
Physical status	1	Clinically healthy
	2	Mild systemic disease
	3	Severe systemic disease
	4	Severe systemic disease that is a constant threat to life
	5	Moribund and not expected to survive more than 24 hours without surgery
	E	Emergency or non-emergency procedure
Sex		Male or Female
Reproductive status before the procedure		Neutered or Intact

### Statistical analysis

Statistical analysis was carried out via a commercially available software (IBM SPSS Statistics for Windows, Version 26.0. Armonk, NY: IBM Corp). If an individual subject had been anesthetized on multiple occasions, only the first anesthetic episode was included in the analysis to avoid carry-over effect. Data were analyzed using multivariate linear regression with backward elimination. Breed was included as an independent variable if there were 40 or more subjects representing that breed. Three models were built. The first used the dose per mass unit of propofol (mg kg^-1^) as the dependent variable and absolute age, breed (for breeds represented by more than 40 subjects), sex, weight, BCS, ASA status, level of sedation, and mg kg^-1^ dose of intramuscular (IM) hydromorphone, morphine, methadone, butorphanol, buprenorphine, acepromazine, dexmedetomidine, midazolam, alfaxalone, ketamine, and sub-cutaneous or IM maropitant, and IV acepromazine, hydromorphone, morphine, methadone, butorphanol, fentanyl, dexmedetomidine, midazolam, diazepam, alfaxalone, ketamine, and lidocaine as independent variables. The second model was identical to the first except percentage of life expectancy was substituted for age as an independent variable. The third model was identical to the first two except breed was excluded as an independent factor, and physiologic age in relation to humans based on body weight, calculated as per a previously published equation [37], was used as the independent variable in lieu of absolute age or elapsed life expectancy. For this model, dogs were divided into weight groups (6.8 kg-13.6 kg, 13.7–22.7 kg, 22.8–34 kg, 34.1–45.3 kg, >45.4 kg) and excluded if their weight was below 6.8 kg. The cut-off age in canine years of age corresponding to 65 years of age of a human subject was identified for each canine weight group based on previously published data [35]. If significance was found, a paired t test was used to test the DOP for dogs within the same weight range between subjects under and above the calculated age cut off (<65 and ≥65 in human years). The DOP for each interquartile of life expectancy (<25%, 25–50%, 50–75%, 75–100%, >100%) was compared using one-way ANOVA. An additional analysis was made using a subgroup of 56 subjects that did not receive premedication drugs before propofol administration, and the DOP from the 20 youngest and the 20 oldest dogs was compared using a 2-way unpaired t-test. Significance was set at α = 0.025.

## Results

The initial selection process generated a sample of 2346 dogs, which was further screened and filtered by a board-certified anesthesiologist for accuracy of data. A total of 1397 dogs were included for the final analysis (Table 2). The sample size included in the elapsed percentage of life expectancy model was *n* = 1276 due to the lack of available data in the literature on the expected life span of certain breeds. Average age and weight (mean ± SD) were 7.2 ± 4.1 years (95% CI: 7–7.4) and 19 ± 14 kg (95% CI 18–20), respectively. Average percentage of elapsed life expectancy of the studied population was 59.8 ± 33% (95% CI: 58–62; based on 1276 subjects). The mean DOP needed to perform endotracheal intubation was 3.7 ± 1.8 mg kg^-1^. A synopsis of the drugs administered in a multimodal anesthetic protocol regimen along with propofol is provided in Table 3. Fifty-six subjects received no drugs other than propofol. There were 101 represented breeds with the most represented being: Boxer (*n* = 46), Chihuahua (*n* = 55), Dachshund (n = 108; long-haired and short-haired combined), German Shepherd dog (*n* = 44), Golden Retriever (*n* = 42), Labrador Retriever (*n* = 185), Maltese (*n* = 54), Pitbull (*n* = 69), Shih Tzu (*n* = 72), Yorkshire terrier (*n* = 56). In addition, subjects of mixed breed (<10kg; *n* = 61. >10kg; *n* = 180) were also largely present. Cut-off ages for the weight groups used in the physiologic age statistical model were 13 (6.8 kg-13.6 and 13.7–22.7 kg), 12 (22.8–34 kg), 11 (34.1–45.3 kg), and 10 (>45.4 kg) years, respectively [35]. Absolute and physiologic ages failed to be significant predictors of DOP in the respective statistical models, whereas percentage of elapsed life expectancy was significant. There was no difference between DOPs from the five life expectancy interquartiles (*P* = 0.20; Table 4), and between youngest and oldest subjects (*P* = 0.86) which received no premedication in this cohort for DOP. Significant predictors for reduced or increased DOP for the three models are presented in Table 5.

**Table 2:** Summary of demographic and perianesthetic data from 1397 dogs. ASA = American Society of Anesthesiologists; BCS = Body Condition Score; CI = Confidence Interval; DOP = Dose of propofol; E = Emergency; MI = Minimally Invasive; SD = Standard Deviation; ST = Soft Tissue Surgery.

	Number of Subjects	Female		Male		Body Weight Mean ± SD (kg)	Age Mean ± SD (years)	Median BCS	Frequency of ASA Status		Frequency of E Status	Frequency of Sedation Scores		Frequency of procedural categories		Mean ± SD DOP (mg kg^-1^)
		Intact	Neut.	Intact	Neut.											
**All Breeds**	1397	136	567	186	508	19 ± 14 (95% CI: 18.3–19.8)	7.2 ± 4.1 (95% CI: 7.0–7.4)	5 (4–6)	I	217	211	0	120	Dental	51	3.7 ± 1.8 (95% CI: 3.6–3.7)
									II	792		1	570	Imaging	527	
									III	326		2	531	MI	45	
									IV	59		3	164	Orthopedic	253	
									V	3				ST	520	

**Table 3:** Synopsis of the drugs administered in a multimodal anaesthetic protocol regimen along with propofol.

Drug	Route	Number of Subjects	Dose (Mean ± SD) mg kg^-1^
Acepromazine	IM	125	0.03 ± 0.03
Acepromazine	IV	3	0.02 ± 0.02
Alfaxalone	IM	36	1.3 ± 0.6
Alfaxalone	IV	4	1.2 ± 0.7
Atropine	IM/IV	20	0.03 ± 0.01
Buprenorphine	IM	3	0.03 ± 0.01
Butorphanol	IM	239	0.34 ± 0.1
Butorphanol	IV	13	0.4 ± 0.2
Dexmedetomidine	IM	361	0.004 ± 0.005
Dexmedetomidine	IV	59	0.002 ± 0.004
Diazepam	IV	14	0.26 ± 0.11
Fentanyl	IV	175	0.004 ± 0.002
Glycopyrrolate	IM/IV	8	0.01 ± 0.01
Hydromorphone	IM	86	0.12 ± 0.05
Hydromorphone	IV	5	0.12 ± 0.04
Ketamine	IM	1	5
Ketamine	IV	119	1.9 ± 1.3
Lidocaine	IV	104	1.4 ± 0.6
Maropitant	SQ/IV	46	1.0 ± 0.04
Methadone	IM	578	0.35 ± 0.13
Methadone	IV	18	0.30 ± 0.11
Midazolam	IM	167	0.22 ± 0.08
Midazolam	IV	262	0.24 ± 0.07
Morphine	IM	190	0.41 ± 0.17
Morphine	IV	6	0.32 ± 0.04

**Table 4:** Summary of the number of subjects and dose propofol for each life expectancy interquartile (*n* = 1276).

Life Expectancy Interquartile	Number of Subjects	Dose of Propofol (mean ± SD) mg kg^-1^	*P*-value
0–25%	249	3.9 ± 2.3	0.20
25–50%	243	3.8 ± 1.8	
50–75%	317	3.6 ±1.8	
75–100%	341	3.7 ±1.7	
>100%	126	3.4 ± 1.6	

**Table 5:** Summary of predictors that increase (+ UC) and decrease (- UC) the dose of propofol for the three statistical models used for data analysis. CI = Confidence Interval; NA = Non-Applicable; NS: non-significant; SD = Standard Deviation; UC = Unstandardized Coefficient. Note: non-significant *P*-values for absolute age and physiologic age are found under the respective models.

Variable	Dose (mg kg ^-1^) Mean ± SD	Model 1 –Absolute Age			Model 2 –Life Expectancy			Model 3 –Physiologic Age		
		UC	95% CI	*P*-value	UC	95% CI	*P*-value	UC	95% CI	*P*-value
(Constant)		4.29	4.0–4.5	<0.001	4.4	4.2–4.7	<0.001	4.4	4.1–4.7	<0.0001
Absolute Age (year)	NA	N/A	N/A	N/A	NA	NA	NA	NA	NA	NA
Life Expectancy (%)	NA	NA	NA	NA	-0.37	-0.65 to -0.08	0.013	NA	NA	NA
Physiologic Age (year)	NA	NA	NA	NA	NA	NA	NA	-0.004	-0.009 to 0.001	.098
Neutered Male	NA	-0.33	-0.5 to -0.1	0.0001	-0.31	-0.5 to -0.1	0.002	-0.3	-0.5 to -0.1	.001
Chihuahua	NA	+1.43	0.9 to 1.9	0.0001	+1.32	0.8 to 1.8	0.0001	+1.4	0.9 to 1.9	.000
Maltese	NA	+1.28	0.8 to 1.8	0.0001	+1.15	0.6 to 1.7	0.0001	+1.3	0.8 to 1.8	.000
Yorkshire Terrier	NA	+2.05	1.6 to 2.5	0.0001	+1.88	1.4 to 2.3	0.0001	+2.0	1.6 to 2.5	.000
Shih Tsu	NA	+0.60	0.2 to 1.0	0.006	NS	NS	NS	+0.6	0.2 to 1.1	.006
Mixed breed <10kg	NA	+1.06	0.6 to 1.5	0.0001	+0.88	0.4 to 1.4	0.0001	+1.1	0.6 to 1.5	.000
Labrador Retriever	NA	-0.40	-0.7 to -0.1	0.006	-0.58	-0.9 to -0.3	0.0001	-0.4	-0.7 to -0.1	.007
Golden Retriever	NA	NS	NS	NS	-0.59	-1.1 to -0.03	0.038	NS	NS	NS
Boxer	NA	-0.58	-1.1 to -0.1	0.029	-0.73	-1.3 to -0.2	0.008	-0.6	-1.1 to -0.1	.030
ASA “E” Status	NA	-0.33	-0.6 to -0.1	0.014	-0.35	-0.6 to -0.1	0.017	-0.3	-0.6 to -0.1	.017
Dental Procedure	NA	NS	NS	0.61	0.1 to 1.1	0.017	NS	NS	NS	NS
IM Butorphanol (mg kg ^-1^)	0.35 ± 0.11	-1.27	-2.0 to -0.5	0.001	-0.76	-1.5 to -0.1	0.034	-0.9	-1.6 to -0.3	.006
IM Dexmedetomidine (μg kg ^-1^)	0.004 ± 0.005	-0.079	-0.1 to -0.05	0.0001	-0.10	-0.1 to -0.6	0.0001	-0.08	-0.1 to -0.05	.000
IV Dexmedetomidine (μg kg ^-1^)	0.002 ± 0.004	NS	NS	NS	-0.32	-0.6 to -0.02	0.037	NS	NS	NS
IM Hydromorphone (mg kg ^-1^)	0.12 ± 0.05	-5.89	-8.8 to -3.0	0.0001	-4.73	-7.7 to -1.7	0.002	-5.2	-8.0 to -2.4	.000
IV Fentanyl (μg kg ^-1^)	0.004 ± 0.002	-0.10	-0.2 to -0.04	0.002	-0.07	-0.1 to -0.003	0.039	-0.09	-0.2 to -0.02	.007
IV Midazolam (mg kg ^-1^)	0.24 ± 0.07	-2.68	-3.6 to -1.8	0.0001	-2.65	-3.6 to -1.7	0.0001	-2.7	-3.6 to -1.7	.000
IV Ketamine (mg kg ^-1^)	1.9 ± 1.3	-0.32	-0.5 to -0.2	0.0001	-0.31	-0.5 to -0.2	0.0001	-0.3	-0.5 to -0.2	.000
IV Lidocaine (mg kg ^-1^)	1.4 ± 0.6	-0.40	-0.6 to -0.1	0.002	-0.43	-0.7 to -0.2	0.002	-0.4	-0.6 to -0.1	.002
IV Alfaxalone (mg kg ^-1^)	1.2 ± 0.7	NS	NS	NS	-2.34	-4.4 to -0.3	0.023	NS	NS	NS

## Discussion

During the process of determining the effective dose of sedatives and anesthetics, patient related factors such as age, sex, and weight are usually considered. In this large retrospective study, we have investigated the hypothesis that the DOP per unit of mass required to perform endotracheal intubation in elderly dogs would not differ from that required for young and adult subjects. Based on our results, we must accept the null hypothesis as true for the models that used raw age as a predictor, as DOP was not affected by absolute and physiologic age. This finding is in striking contrast with what occurs in people [18, 20, 38], in which a significant reduction in DOP occurs in the elderly after 60 years of age. This phenomenon has been attributed to pharmacokinetic and pharmacodynamic differences of juvenile and adult individuals, such as a reduction in central compartment and clearance [39], and increased sensitivity to the hypnotic effects of propofol [15, 17, 40] in the elder. These pharmacokinetic and pharmacodynamic changes have also been observed in a study with a limited canine population of geriatric dogs, in which a lower induction dose and a slower clearance of propofol were observed [24]. Based on absolute and physiologic age, our results are also in contrast with those from Faggella and Aronsohn, in which young and healthy dogs required a larger induction and maintenance dose of propofol compared with adult dogs [41]. In fact, absolute age is not a reliable predictor of perianesthetic mortality in dogs. However, other factors such as obesity may contribute to the absolute age at death. For example, a study found that median age at death was negatively correlated with average ideal bodyweight with 40% variability in age at death explained by bodyweight in 81 breeds [31]. This seems to reinforce the idea that dogs should be used carefully as aging models in pharmacokinetic and pharmacodynamic studies involving propofol, and potentially other drugs.

Percentage of elapsed life expectancy was a statistically significant predictor of DOP. The interpretation of the significant unstandardized coefficients should be carried out by attributing a decrease (-UC) or an increase (+UC) of the parameter of interest by the unit of measure of that parameter. The interpretation of the statistical model predicts a decrease of DOP by 0.37 mg kg^-1^ for each 100% change in life expectancy. In other words, a dog at 1.2 times their life expectancy would need 0.37 mg kg^-1^ less than a dog at 0.2 times their life expectancy, which represent a clinically unimportant effect.

We rejected the null hypothesis stating that none of the investigated variables in this cohort would constitute a predictor of DOP as several predictors were identified. Likely not coincidentally, subjects from several small breeds, Yorkshire, Maltese, Chihuahua, Shih Tzu, and mixed-breed dogs under 10 kg have been found to have increased requirements of propofol for induction of anesthesia, whereas large breeds such as Boxer, Golden Retriever, and Labrador Retriever have been found to require a lower DOP. This finding has never been documented previously on a large sample of dogs but is not surprising. Evidence to support these findings is provided by Martinez *et al*. who demonstrated that drug clearance is not directly proportional to body weight of dogs of different breeds, and that pharmacokinetics of a drug could markedly differ in large or small dogs from concentrations estimated from studies using beagle-sized dogs as their model [42]. Furthermore, smaller dogs have been shown to have increased mass-specific metabolic rate and higher metabolically active organs, whereas larger breeds have significantly smaller liver, kidney, and brain relative size compared to smaller breeds [43, 44], which could explain differences in mass specific metabolism based on dog size [43], independent of BCS. Furthermore, potential breed and sex differences in propofol hydroxylation by CYP2B11 could be resulting from differences in the liver content of this CYP, although these conclusions were derived from a limited sample size, age (1–3 year of age), and body weight (15–31 kg) [45]. Propofol disposition may be different in Greyhound dogs compared to mixed-breed dogs with increased recovery time [46, 47]. This cohort included only 3 Greyhound dogs; therefore, data is insufficient to detect any statistical difference with other breeds. Based on our results, we recommend increasing the calculated DOP by 2, 1.4, 1.3, 1.1, and 0.6 mg kg^-1^ for Yorkshire Terrier, Chihuahua, Maltese, mixed breed dogs under 10 kg, and Shih Tzu subjects, respectively, and to reduce it by 0.58 to 0.73 mg kg^-1^ in Boxer, 0.6 mg kg^-1^ in Golden Retriever, and 0.4 to 0.58 mg kg^-1^ in Labrador Retriever.

In our analysis, the status of neutered male has been found to be a significant predictor of reduced DOP by 0.3 to 0.33 mg kg^-1^ compared to other sex status depending on the statistical model. This is in contrast with the conclusions from other studies [41, 45, 48, 49] in which male dogs required a slightly higher induction dose than female dogs. However, studied samples included intact and gonadectomized subjects, so the role of the reproductive status on these results is difficult to determine. The available information on the role of gonadectomy in the development of obesity in dog is conflicting [50, 51].

The emergent nature of the anesthetic procedure was significant predictor for reduced DOP. The average decrease in DOP for patients assigned an ASA E in the present study ranged from 0.3 to 0.35 mg kg^-1^ when compared to non-E ASA patients. It has been speculated that critically ill patients may require lower doses of induction agents due to their compromised health status and decreased mentation [52]. However, the impact of this dose reduction on hemodynamic and respiratory performances in critically ill dogs is unknown, and DOP should always be tailored to the individual subject and to their health status.

As reported previously in the literature [48, 53, 54], several premedication drugs reduced DOP. In the context of this analysis, the reader should be advised to interpret the UCs in the following manner: “For each mg of each drug used, a linear decrease (-UC) or increase (+UC) in mg kg^-1^ of propofol is predicted”. In this cohort of dogs, some drugs administered intramuscularly prior to induction reduced DOP. Only two opioid compounds used intramuscularly reduced DOP: butorphanol and hydromorphone. While the sparing effect on DOP of butorphanol was modest (0.76 to 1.27 mg kg^-1^ per mg used), hydromorphone produced a decrease in DOP by 0.47 to 0.59 mg kg^-1^ per 0.1 mg kg^-1^, which is a commonly used dose. We speculate that the lack of sparing effect by other intramuscular opioids and the greater observed sparing effect of hydromorphone are due to its higher potency. The alpha_2_-adrenergic agonist dexmedetomidine also exerted a significant sparing effect as it reduced DOP when administered both intramuscularly and IV, as reported in previous publications [53].

The technique of co-induction consists in the practice of administering one or more opioid, sedative, hypnotic, or dissociative compounds to reduce the dose of the main anesthetic employed [54]. In this cohort, IV fentanyl, midazolam, ketamine, lidocaine, and alfaxalone administration alone with propofol, or in various combinations with other co-induction agents, reduced DOP, which agrees with the existing literature. Notably IV midazolam in the context of the mean dose used in this study (0.24 mg kg^-1^), produced a reduction in DOP by 2.65 to 2.7 mg kg^-1^ of propofol. The magnitude of this sparing effect is greater than previously reported, which ranged from 17.6 to 42.1% (1.9 mg/kg to 1.1 mg/kg with 0.3 mg/kg of midazolam in ill dogs, and from 3.4 mg/kg to 2.8 mg/kg with 0.2 mg/kg of midazolam in healthy dogs, respectively) [52, 55]. We speculate that some pre-analytical bias may have occurred in this cohort, as midazolam is often prescribed to juvenile or elderly dogs, or those that present with co-morbidities and may be severely ill. This decisional bias may cause the reduction of DOP seen with midazolam appear greater than what it would be if the same dose was used on a healthy population.

Interestingly, weight, BCS, and level of sedation were not found to be a predictor of DOP. The lack of reduction in DOP with increasing weight is in contrast with what observed in human medicine [56]. In fact, adjustments in the DOP based on lean body weight for obese patients have been implemented for people [56, 57]. In veterinary medicine, a prospective clinical study investigating the effect of body condition on propofol requirements in dogs, overweight (BCS = 6/9) and obese dogs (BCS (7-9/9) have been found to require a lower DOP (1.83 ± 0.36 mg kg^-1^) compared to dogs with normal body condition (2.24 ± 0.53 mg kg^-1^; Boveri et al., 2013). Subjects included (ASA I-III), premedication regimen, and propofol delivery technique were standardized in this prospective study [58]. In contrast, our results reflect a much larger population of dogs, with a wider variety of health status, comorbidities, and preanesthetic regimen. These factors may have contributed to these contrasting results. In the present study, the effect of body condition, actual body fat composition, and pure effect of BCS on the UCs are unknown. Therefore, based on our results, we do not recommend adjusting DOP in overweight and obese subjects, but based on breed.

Several limitations to this study should be considered. This study is retrospective in nature and, as such, carries the inevitable risk of inaccuracies and inconsistencies in the medical and anesthetic records. However, significant attempts at reducing bias by strict filtering of incomplete records by the same boarded anesthesiologist (CEH) were made in addition to the routine triple check-point system used as a standard operating procedure at this anesthesiology service. Different combinations of sedative agents have been used in this study, and it is important to note that the effects of time of administration and peak of sedation of these sedatives on DOP is unknown. Since multiple individuals administered propofol during induction, bias may have occurred due to inconsistent speed of injection, or due to excessive administration of propofol before waiting the proper amount of time for attempting intubation which have been shown to affect DOP [59]. Breed classification was based on the phenotype of the subject and not on DNA profiling or pedigree certificate from the American Kennel Club; Therefore, misclassification of subjects based on breed may have occurred. Finally, the pharmacodynamic effects of adjusting DOP based on the coefficients determined in this study are unknown.

In conclusion and in contrast to what is observed in people, an age cut-off predictive of DOP does not exists in dogs. Only percentage of elapsed life expectancy along with other factors such as breed, premedication drug, emergency procedure, and reproductive status significantly altered DOP. Based on these findings, absolute age can be excluded as a confounding factor determining DOP. In contrast with absolute age, elapsed life expectancy may hide unexpected value as a predictor and further research incorporating it in future study designs is warranted. This information is relevant not only to researchers, but to the veterinary practitioner who routinely uses propofol for induction of general anesthesia as adjustments in the amount of propofol drawn should be made based on significant predictors of DOP such as breed, premedication drugs, reproductive and emergency ASA status and not based on absolute or physiologic age. Although the reduction in DOP was statistically significant in the model accounting for percentage of life expectancy elapse at the time of the anesthetic episode, this has minor clinical importance as this reduction was minimal. As the magnitude and incidence of the post-induction side effects of propofol in clinical practice is unknown, we emphasize the practice of individualizing the calculated induction dose based on a holistic approach that includes these predictive factors along with the overall health status of the canine patient to minimize anesthetic risk and optimize perianesthetic outcome.

## Supporting information

S1 Data (XLSX)
